# Copper, Iron, and Manganese Toxicity in Neuropsychiatric Conditions

**DOI:** 10.3390/ijms22157820

**Published:** 2021-07-22

**Authors:** Beata Tarnacka, Anna Jopowicz, Maria Maślińska

**Affiliations:** 1 Department of Rehabilitation Medicine, Faculty of Medicine, Warsaw Medical University, Spartańska 1, 02-637 Warsaw, Poland; 2 Department of Rehabilitation, Eleonora Reicher National Institute of Geriatrics, Rheumatology and Rehabilitation, Spartańska 1, 02-637 Warsaw, Poland; annajopowicz@gmail.com; 3 Department of Early Arthritis, Eleonora Reicher National Institute of Geriatrics, Rheumatology and Rehabilitation, Spartańska 1, 02-637 Warsaw, Poland; maslinskam@gmail.com

**Keywords:** copper, iron, manganese, neurodegeneration

## Abstract

Copper, manganese, and iron are vital elements required for the appropriate development and the general preservation of good health. Additionally, these essential metals play key roles in ensuring proper brain development and function. They also play vital roles in the central nervous system as significant cofactors for several enzymes, including the antioxidant enzyme superoxide dismutase (SOD) and other enzymes that take part in the creation and breakdown of neurotransmitters in the brain. An imbalance in the levels of these metals weakens the structural, regulatory, and catalytic roles of different enzymes, proteins, receptors, and transporters and is known to provoke the development of various neurological conditions through different mechanisms, such as via induction of oxidative stress, increased α-synuclein aggregation and fibril formation, and stimulation of microglial cells, thus resulting in inflammation and reduced production of metalloproteins. In the present review, the authors focus on neurological disorders with psychiatric signs associated with copper, iron, and manganese excess and the diagnosis and potential treatment of such disorders. In our review, we described diseases related to these metals, such as aceruloplasminaemia, neuroferritinopathy, pantothenate kinase-associated neurodegeneration (PKAN) and other very rare classical NBIA forms, manganism, attention-deficit/hyperactivity disorder (ADHD), ephedrone encephalopathy, HMNDYT1-SLC30A10 deficiency (HMNDYT1), HMNDYT2-SLC39A14 deficiency, CDG2N-SLC39A8 deficiency, hepatic encephalopathy, prion disease and “prion-like disease”, amyotrophic lateral sclerosis, Huntington’s disease, Friedreich’s ataxia, and depression.

## 1. Introduction

Copper (Cu), iron (Fe), and manganese (Mn) play important roles in brain biology. They are present in some regions of the brain in very small millimolar concentrations. Cu and Fe are involved in the production of oxygen radicals; therefore, they are major causes of oxidative stress. The above-mentioned metals may also have an impact on protein misfolding and the progression of the neurodegenerative processes. Metals are essential for their integral roles in many enzymes that catalyse metabolic or biochemical processes common to all life forms.

The crossing of metals through the blood–brain barrier (BBB) is very strictly regulated. Enzymes, transporters, and chaperones regulate the metal ion content within the brain. In healthy people, the concentration of free metal ions is very low. Metal ions are selectively delivered where their action is needed. Metal dyshomeostasis is widely documented as a cause of several neurodegenerative diseases, including prion disease, Alzheimer’s disease, Parkinson’s disease, amyotrophic lateral sclerosis (ALS), and Huntington’s disease (HD), among others [1,2,3,4,5]. Metal ions play a key role as necessary elements for biological processes and act as cofactors for many enzymes, but they can also be potentially dangerous to a cell involved in redox reactions that lead to the formation of reactive oxygen species. Under normal conditions, reactive oxygen species (ROS) are detoxified by the cell, but in pathological states, ROS production exceeds the capabilities of intracellular antioxidant defense, and an increase of ROS production is observed.

This is a condition known as oxidative stress. Various studies have expanded on conceivable molecular mechanisms and signaling pathways through which metals cause neurotoxicity and degeneration of the central nervous system [6]. A number of explanations of cell death due to metal neurotoxicity have been proposed. These include malfunction of the mitochondria and interruption of cell energy metabolism, oxidative stress, as well as modification in levels of neurotransmitters and excitotoxic cell death [6].

Oxidative stress, described as the creation of ROS, is a focus point of many other mechanisms of metal toxicity. Furthermore, the occurrence of metal toxicity gives rise to neuronal damage. Mitochondria are intracellular objects for metals toxicity. The resulting oxidation of membranal polyunsaturated fatty acid creates lipid peroxides (LPO), thus affecting mitochondrial permeability and inducing cell apoptosis [6,7,8].

Additionally, proteins associated with neurodegenerative diseases, such as amyloid-β (Aβ), α-synuclein (αS), and prion protein (PrP), bind several metal ions that can affect the process of aggregation of Aβ and αS, for example, in Parkinson’s disease or Alzheimer’s disease. In those cases, Cu and Fe generate reactive oxygen species (ROS) through Fenton reactions “(Cu^1+^ + H_2_O_2_ → Cu^2+^ + OH**·** + OH^−^ or Fe^2+^ + H_2_O_2_ → Fe^3+^ + OH**·** + OH^−^)”. This process may lead to oxidative stress because of the formation of the highly reactive hydroxyl radical (OH**·**) in the substantia nigra [9].

In the following review, Cu, Fe, and Mn rare storage diseases with neuropsychiatric manifestations will be discussed. These rare disorders were selected to be presented in this review because they represent a combination of neurological and psychiatric signs and additionally due to their rarity, which may pose diagnostic difficulties. The current review can assist clinicians in the differential diagnosis. This review will also present the new methods of its treatment and a brief pathophysiological profile.

## 2. Copper Toxicosis

Copper is a heavy metal that plays an essential role in many physiological processes, such as skin pigmentation, myelination, Fe homeostasis, oxygen metabolism, and the synthesis of neurotransmitters. Copper is essential in a variety of biological processes and plays an important role as a cofactor or as a structural component in numerous cuproproteins. The oxidative state of copper may change from Cu(I) to Cu(II). Copper can also interact with ceruloplasmin (Cp) and takes part in Fe homeostasis, exhibiting Cu-dependent oxidase activity, with Fe(II) into Fe(III) transformation taking part in Fe transport in the plasma [10]. Copper accumulates mostly in the liver and brain, which is the main organ responsible for its metabolism, and it enters the bloodstream via the protein adenosine triphosphatase 7A (ATP7A). Adenosine triphosphatase (ATPase) 7B (ATP7B) is also responsible for balancing Cu levels in tissues; ATP7B is involved in Cu incorporation into apoceruloplasmin and for the synthesis of functional ceruloplasmin, and this enzyme also facilitates the excretion of biliary Cu [11]. Copper is incorporated into the blood of ceruloplasmin molecules, and physiologically, Cu is not present in the free form. As mentioned before, Cu is also stored in the brain, especially in the *substantia nigra*, hippocampus, cerebellum, olfactory bulbs, hypothalamus, and cortex [12]. Excess copper may cause neurodegeneration [12]. Wilson’s disease (WD) is a good example of this process.

### 2.1. Neuropsychiatric Diseases Associated with Copper Toxicosis

#### Wilson’s Disease

Wilson disease is an inherited Cu metabolism disorder with Cu accumulation in many organs, particularly the liver and brain. The disease is caused by mutations in the ATP7B gene, which encodes a transmembrane Cu-transporting ATPase. The pathological highly toxic excess of Cu (“free” copper) released from hepatocytes into the bloodstream and then to the brain causes a wide range of neuropsychiatric symptoms. After crossing the BBB, copper is stored by astroglia, causing oedema and degeneration; it is believed that neurons are affected by the functional insufficiency of astroglia [13]. In vitro studies have shown that the administration of Cu significantly reduces the survival of neurons [14]. This ion damages the regulation of glutamate by triggering NMDA receptors and activating the excitotoxic cascade that produces nitric oxide (NO) by stimulating the expression of nitric oxide synthase (NOS1-3). Ceruloplasmin deficiency with excess free Cu and Fe deposits can lead to excitotoxicity, enhanced nitrosative/oxidative stress, and damage to mitochondria [12]. There are also hypotheses that ceruloplasmin can play a protective role in neurodegenerative diseases [15]. Wilson’s disease mostly presents with hepatic disturbances; the disease is manifested by neurological signs in 40–50% of cases and by psychiatric signs in 10–25% [16]. The most common psychiatric disturbances are personality disorders, including abnormalities, antisocial behaviour, disinhibition, and irritability. Mood disorders such as bipolar disorders or depression with suicidal attempts are also commonly diagnosed [17]. Psychosis and other psychiatric alterations, such as anorexia and sleep disturbances, can be seen [17]. When psychotic symptoms occur as the first manifestation of WD, they can result in diagnostic and therapeutic challenges, but such manifestations must be taken into consideration [17]. The neurological presentation is associated with dysarthria (extrapyramidal, dystonic, cerebellar, mixed, or unclassified origin) or other speech disorders, dysphagia, salivation, involuntary movements such as tremor (“wing beating”), dystonia, athetosis, chorea, parkinsonism, cerebellar ataxia (gait ataxia), gait and balance disturbances, and cognitive decline [18].

The diagnosis of WD is based on the presence of Kayser–Fleischer rings (brown discoloration of the cornea), which is the pathognomonic sign of this disease. In WD, the concentration of Cu in the brain in the liver is high, but in blood, low levels are diagnosed. Levels of urinary Cu are typically increased and are used as a biomarker of the disease, and ceruloplasmin is also decreased Cu deposits in the brain (appearing as hypointense lesions in T2-weighted MRI imaging and hyperintense in T1-weighted imaging) occur as a result of the paramagnetic qualities of copper and are mainly visible in the globus pallidus, putamen, caudate nuclei, and substantia nigra. Other changes appearing as hyperintense lesions in T2-weighted MR imaging are associated with cellular oedema, necrosis, cystic degeneration, and glial proliferation; these are mostly visible in the putamen, caudate, thalami, pons, midbrain, white matter, and cerebellum [16].The disease is mostly successfully treated with medications (chelators, zinc (Zn) sulfate). It is currently recommended by international societies that chelators should be the first-line treatment of WD [16]. The most prominent goal for new treatment strategies is to prevent neurological deterioration during treatment. From the current new therapeutic strategies for WD treatment, bis-choline tetrathiomolybdate and once-daily trientine are the most advanced and promising methanobactin bis-choline in animal models [19]. The gene therapy will also be an option for the treatment of WD in the coming years. Selective serotonin reuptake inhibitors can be chosen as a first-line treatment for depression in these patients. For manic or hypomanic syndromes in the treatment of Parkinson’s disease with psychotic symptoms, clozapine or quetiapine monotherapy is recommended due to the relatively low risk of exacerbating parkinsonism [20]; olanzapine could be another therapeutic option. Behavioral disturbances can also be treated with quetiapine and tiapride [20].

## 3. Iron Toxicosis

Brain neurobiology and homeostasis is a complicated mechanism. Ferrous iron (Fe^2+^) is transported out of the endothelial cell into the interstitial fluid by ferroportin. Fe^2+^ in the intestinal fluid can be oxidized to Fe^3+^ by membrane-bound ceruloplasmin or hephaestin, and by soluble ceruloplasmin secreted from pericytes and astrocytes. Ferritins are Fe storage proteins, storing ferric iron (Fe^3+^) in a water-soluble bioavailable form. Hemosiderin is formed after the degradation of ferritin within lysosomes and mostly contains Fe^3+^. Iron is more easily released from hemosiderin in contrast to ferritins. Hemosiderin is formed mostly during iron overload and accumulates in neurons and glia in aging and in pathological conditions as in patients with Alzheimer disease, Parkinson’s disease, progressive supranuclear palsy, and other neurodegenerative disorders [21]. Neuromelanin efficiently may bind Fe in neurons. Neurons take up Fe, essentially from transferrin through transferrin-receptor (TfR)-mediated endocytosis, and export excess Fe via ferroportin. In most cells of the body, Fe homeostasis is regulated at the level of the translation or stabilization of mRNAs by iron-regulatory proteins and their regulation of TfR1 and ferritin [21].

In vitro and in vivo experiments have shown that cellular oxidative stress can be induced by Fe overload and cause increased lipid peroxidation as well as protein and nucleic acid modifications [22,23,24]. As mentioned before, Fe, similar to Cu, is a redox-active metal occurring in two main oxidation states, +2 and +3. The interactions between Fe and respiration products in an aerobic state during the Fenton reaction lead to free hydroxyl radical formation [4,25].

Iron overload can also result in mitochondrial dysfunction [4,25]. An Fe excess may also change the mitochondrial morphology and decrease their mitochondrial membrane potential, leading to decreased ATP production [26]. Sripetchwandee et al. in their study observed a dose and time-dependent mitochondrial swelling, depolarisation, and ROS production as a result of ferrous and ferric iron (Fe, +2) overexposure [27]. The authors also discovered that in rat models, a mitochondrial calcium uniporter blocker could completely prevent ROS production and mitochondrial depolarisation [27].

In recent literature, several models of Fe toxicity were proposed, including also an inflammation model that initiates an intercellular signaling cascade with microglia activation and astrocytes stimulation to release hepcidin which, in turn, serves as a signal to neurons to prevent Fe release, which could result in neuronal death [28]. In the normal adult brain, microglia are in a nonactivated state, displaying a ramified morphology. After the detection of abnormalities, an amoeboid microglia phenotype is formed and leads to the release of both pro- and anti-inflammatory cytokines [29].

In recent years, Fe homeostasis has been better known; however, many questions remain regarding how the brain regulates Fe storage in neurons, astrocytes, and oligodendrocytes. In healthy men, Fe deposition depends on areas of the brain where it is stored, and higher concentrations in the substantia nigra, the globus pallidus, thalamus, dentate gyrus, ventral pallidus, and red nucleus are seen. It is commonly known that the brain requires high levels of Fe and a constant supply of oxygen. Therefore, it is critical to maintain the optimal Fe levels for its proper function. Iron is involved in several brain processes, such as myelin synthesis and the regulation of brain neurotransmitters. Iron can act as a cofactor of enzymes for the synthesis of dopamine, serotonin, and cholinergic neurotransmitters [25]. It plays roles in emotion, cognitive, and motor function. Despite the important roles mentioned above in the brain, Fe overload can induce neurotoxicity [24]. This process has been demonstrated in various neurodegenerative disorders and in normal ageing [4,30]. Aging is associated with an increased accumulation of iron in the brain. Zecca et al. measured the levels of iron, copper, and their major molecular forms such as ceruloplasmin, neuromelanin, manganese-superoxide dismutase (SOD), and copper/zinc-SOD in the substantia nigra and locus coeruleus (LC) in normal subjects at different ages [31]. They found in LC lower Fe levels in comparison with levels in substantia nigra (SN), and the ratio of heavy-chain ferritin/iron in the LC was higher than in the (SN). Copper and ceruloplasmin levels were similar in both regions. Manganese-SOD and copper/zinc-SOD also had similar age trends in LC and SN [31].

Elevated brain Fe in elderly people may trigger brain dysfunction and concomitant cognitive decline. However, the exact mechanism underlying the deleterious impact of Fe on brain function in ageing is unknown [30]. Perhaps these divergent results may be due to the age of the studied patients, as it has been shown that higher Fe levels may be beneficial in younger people but harmful in old age groups [24,25]. In fMRI, higher striatal Fe was linked to greater frontostriatal activity in younger adults and reduced activity in older adults [30]. The authors stated that Fe accumulation can trigger neuroinflammation, which can lead to disrupted frontostriatal activation and working memory decline in older age groups [30]. 

Measuring the amount of nonheme Fe in the brain can lead to a better understanding of the disease pathophysiology and progression as well as improve ability to predict outcome. A detailed summary of earlier work measuring brain Fe content and regional distribution of selected Fe compounds, as well as MRI parameters, has been published by Haacke et al. [32]. The authors proposed several methods for quantitative studies of Fe content. Recently (in the past decade), the new magnetic resonance imaging techniques were developed as quantitative susceptibility mapping (QSM), which enabled investigations for the more comprehensive iron distribution measurement methods in the brain. The number of studies concerning QSM is still limited in regard to most neurodegenerative diseases, but there is growing evidence that suggests it could be a promising tool in the investigation of neurodegeneration [32,33].

Several diseases that present during adolescence in association with Fe overload are presented below.

Neurodegeneration with brain iron accumulation (NBIA) syndromes mostly have an onset in childhood, but some of them present in adolescence or early adulthood. This group of syndromes is related to mutations in pantothenate kinase-associated neurodegeneration (35–50%; *PANK2* gene) and *PLA2G6*-associated neurodegeneration (PLAN; ≈20%; *PLA2G6* gene), followed by mitochondrial membrane protein-associated neurodegeneration; (MPAN 6–10%; *C19ORF12* gene) and β-propeller-associated neurodegeneration (BPAN; 1–2%; *WDR45* gene); fatty acid hydroxylase-associated neurodegeneration (FAHN; *FA2H* gene), neuroferritinopathy (NF; *FTL* gene), aceruloplasminaemia (*CP* gene) and Woodhouse–Sakati syndrome (*DCAF17* gene), Kufor–Rakeb syndrome (*ATP13A2* gene), and COASY protein-associated neurodegeneration (CoPAN; *COASY* gene) [30]. They usually present with predominant bulbar and axial dystonia with spasticity. The presentation of those rare disorders should be included in this review because they feature a combination of dystonia and other neurological symptoms along with psychiatric signs. Unfortunately, they are usually not considered in the differential diagnosis of late-onset isolated (idiopathic) dystonia or psychiatric features by psychiatrists. We present genetically proven cases of NBIA (including aceruloplasminaemia, neuroferritinopathy, pantothenate kinase-associated neurodegeneration) and other syndromes with late onset.

### 3.1. Neuropsychiatric Diseases Associated with Iron Toxicosis

#### 3.1.1. Aceruloplasminaemia

Aceruloplasminaemia is a very rare inherited neurodegenerative disorder associated with systemic Fe overload. It is caused by ceruloplasmin deficiency due to lack of ceruloplasmin ferroxidase activity caused by mutations in the ceruloplasmin gene (70 mutations). A novel homozygous mutation of the ceruloplasmin gene in exon 6 (c.1192–1196del, p. Leu398Serfs) was recently found [34]. Pathological studies have shown that patients with aceruloplasminaemia have severe Fe deposition mostly in astrocytes but also in nerve cells in the basal ganglia, thalamus and cerebellum, with neuronal loss in those regions [35]. In MRI, extensive symmetrical abnormal susceptibility on T2-weighted imaging involving the basal ganglia, thalamus, dentate nuclei, and cortex has been observed [34]. The clinical manifestations of this disease are anaemia, retinal degeneration, diabetes mellitus, and neurological symptoms. Neurological symptoms usually occur after 40 years of age, predominantly as cerebellar ataxia that mostly affects the upper limbs; clinician manifestations include gait ataxia, dysarthria, involuntary movements, dystonia (blepharospasm, grimacing, neck dystonia), chorea and tremor, rigidity, and parkinsonian signs. Cognitive dysfunction is diagnosed in 60% of patients, with a predominance of apathy, depression, and forgetfulness [35]. The diagnosis of aceruloplasminaemia is based on the demonstration of the complete absence of serum ceruloplasmin and neuroimaging, suggesting Fe overload. The above-mentioned laboratory findings include microcytic anaemia, a decreased serum Fe level, and an increased serum ferritin concentration of more than 1000 ng/mL. In T1- and T2-weighted brain magnetic resonance imaging (MRI), hypointensity of the globus pallidus, putamen, caudate, dentate nucleus of the cerebellum, and thalamus was observed [35,36]. Recently, a new method of mapping the Fe distribution in the human brain was invented. The method is based on the measurement of the transverse relaxation rate and water content in a high-field MRI at 4.7 T and revealed Fe accumulation in almost all brain regions [37]. Treatment strategies based on decreasing Fe content by Fe chelation therapy have been applied in some patients with the intravenous administration of deferoxamine or an oral Fe chelating agent, deferasirox, which led only to a mild clinical improvement [38,39]. Iron-chelating therapy is not effective on brain Fe overload because deferoxamine is not able to cross the BBB. Treatment with oral zinc sulfate therapy (200 mg/day) also showed effectiveness in resolving extrapyramidal and cerebellar symptoms by inducing metallothionein synthesis [40]. It was suggested that the combination of an Fe chelator and Zn sulfate may reduce neurological symptoms [35]. Antibiotics, such as tetracyclines, are able to cross the BBB and have Fe-chelating features, and minocycline has been reported as a therapy to block the progression of neurologic signs [41]. A new therapeutic method—administration of ceruloplasmin in animal models—was found [42]. The authors stated that ceruloplasmin is able to cross the BBB, inducing partial restoration of the ceruloplasmin levels a few months later, with brain ferroxidase activation and amelioration of motor incoordination [42].

#### 3.1.2. Neuroferritinopathy

Neuroferritinopathy (NF) is a very rare autosomal dominant syndrome of neurodegeneration previously classified as neurodegeneration with brain iron accumulation type 2, predominantly within the basal ganglia. It was discovered in northeastern England, but some cases were also reported in France, Portugal, the United States, Japan, and Australia [43]. NF is caused by mutations in the ferritin light chain 1 gene (FTL1), which later cause disruption of the ferritin light-chain protein, resulting in an abnormal configuration of the ferritin molecule. Clinically, the disease is characterised by a slowly progressive middle-aged (approximately 40 years old) adult-onset movement disorder often associated with a cognitive and neuropsychiatric phenotype. Neurological symptoms include chorea, dystonia, tremor, parkinsonism, cerebellar ataxia, writer’s cramp, blepharospasm, oromandibular dyskinesia, hypomimia, bradykinesia, and dysphagia.

Cognitive features showed mostly mild defects in verbal fluency. Keogh et al. performed an analysis of 12 patients who had undergone cognitive assessment (Addenbrooke’s cognitive examination or neuropsychometric testing) and found that patients had abnormalities ranging from verbal learning defects to psychiatric abnormalities, including psychosis [43]. It is worth emphasising that neuropsychiatric symptoms began early within 5 years of the onset of motor symptoms.

The diagnosis of NF can be made using a combination of low serum ferritin, MRI, and genetic testing.

McNeill et al. performed an extensive MRI analysis of the neuroradiological features of NF and found in T2-weighed MRI imaging widespread hypointensity in the cerebral cortex, globus pallidus, putamen, caudate nuclei, thalamus, substantia nigra, and dentate nuclei, reflecting the likely central cavitation surrounded by Fe deposition on T2-weighted MRI [36,44,45]. In two cases of NF, the radiological “eye of the tiger sign” characteristic of pantothenate kinase-associated neurodegeneration was seen [36,45]. At present, no proven treatment for neuroferritinopathy is available.

Some attempts at Fe depletion were performed with desferrioxamine (4000 mg weekly for 14 months) and deferiprone (2 g three times a day for 2 months), but all these treatments produced profound, temporary Fe depletion with no significant short-term clinical benefit and no long-term effect [46]. In symptomatic treatment, botulin toxin for focal dystonia and tetrabenazine for chorea with sulpiride and benzhexol for hyperkinetic movements and parkinsonism can be used [31].

#### 3.1.3. Pantothenate Kinase-Associated Neurodegeneration (PKAN) and Other Very Rare Classical NBIA Forms

Pantothenate kinase-associated neurodegeneration is the most frequent NBIA and is also hereditary and progressive. The disease was previously known as Hallervorden–Spatz syndrome, but the name was changed because of unethical activities by authors (Hallervorden and Spatze) in Nazi Germany. Since the identification of PANK2 autosomal recessive mutations causing pantothenate kinase-associated neurodegeneration comprising approximately 50% of NBIA cases, the PKAN prevalence is approximately one to three in 1,000,000 [47]. Pantothenate kinases are enzymes needed for the coenzyme A (CoA) biosynthesis pathway. Classic PKAN manifests in the first decade as progressive dystonia with spasticity and dysarthria. The minority of patients manifest later than 10 years with a more gradual progression, including speech problems such as dysarthria with palilalia and tachylalia [47]. Late presentation is characteristically correlated with cognitive impairment and psychiatric signs such as impulsivity, depression, aggression, and emotional lability [30,47]. In the majority of PKAN cases, abnormalities in the MRI in the globus pallidus and substantia nigra are seen, with a typical “eye of the tiger” sign, but in late-onset cases, this sign can be very subtle; some cases of PKAN showed the involvement of the dentate nuclei (on T2 and Fast spin echo—FSE). In infantile neuroaxonal dystrophy (INAD), hypointensity of the globus pallidus and substantia nigra was observed in all cases, with dentate hypointensity only on T2-weighted images [30]. An effective pharmacological treatment is not yet available for PKAN. Deferiprone, an Fe chelator able to cross the BBB, was administered, but motor symptoms such as dystonia failed to improve only in patients with atypical presentation [48]. In patients with slower progression, some benefits were seen [48]. In the latest trials, different compounds have been tested to increase coenzyme A (CoA) levels as alternative CoA precursors [49]. By the guidelines for medical PKAN treatment options, baclofen, clonazepam, trihexyphenidyl, botulinum toxin, or deep brain stimulation can be used to improve focal dystonia [50].

PLA2G6-associated neurodegeneration (PLAN) is another NBIA syndrome characterised by atypical forms with adolescence or early adulthood onset with dystonia-parkinsonism, pyramidal signs, cerebellar ataxia, cognitive decline, and psychiatric features. On MRI, hypointensity of the globus pallidus and substantia nigra is seen [30]. Mitochondrial membrane protein-associated neurodegeneration (MPAN) can also be diagnosed in early adulthood. Patients have prominent neuropathy and may also present with spastic para- or tetraparesis with muscle atrophy, parkinsonism, dystonia, optic atrophy, dementia, and other psychiatric symptoms. Hypointensity of the globus pallidum and substantia nigra are seen in MRI T2 scans. Β-Propeller-associated neurodegeneration (BPAN) in adulthood presents with dystonia-parkinsonism and dementia, seizures, and ataxia. Hypointensity of the globus pallidus and substantia nigra in MRI scans is also observed, and a T1-hyperintense “halo” signal with a central band of hypointensity in the substantia nigra seems to be a specific finding in BPAN [30]. Fatty acid hydroxylase-associated neurodegeneration (FAHN) in childhood presents with gait disturbances caused by spastic paraplegia, ataxia, and dystonia. Very rare Kufor–Rakeb syndrome is diagnosed in adolescence and is characterised by juvenile parkinsonism, dystonia, autonomic dysfunction, eye movement abnormalities, psychiatric features, and dementia, with hypointensity in the basal ganglia (specifically, the putamen and caudate) in MRI scans [30]. Treatment is mainly symptomatic in those neurodegeneration syndromes; in Kufor–Rakeb syndrome, the combination of levodopa and carbidopa can be used [30].

#### 3.1.4. Friedreich’s Ataxia (FRDA)

Friedreich’s ataxia (FRDA) is an autosomal recessive spinocerebellar ataxia. In most cases, the disease is caused by a homozygous GAA triplet repeat expansion in the frataxin (*FXN*) gene, and the shorter repeat expansion length correlates with age at onset and disease severity [51]. Frataxin is a small, highly conserved protein that is set in the nucleus and expressed in the cytoplasm in the form of a precursor polypeptide that is then transferred into the mitochondria. Preliminary research using yeast demonstrates a strong correlation between frataxin and mitochondrial Fe. This was established by the occurrence of Fe overload and susceptibility to oxidative stress when the frataxin homologue (Yfh1p) is deficient [52]. The frataxin deficiency impedes ISC (iron-sulfur cluster) biogenesis, interrupts mitochondrial Fe homeostasis, and greatly increases sensitivity to oxidative stress. These processes result in progressive cell toxicity and death in the heart, nervous system, and pancreatic β cells [53].

Axonal dystrophy with axonal spheroid formation due to Ca^2+^ imbalance and oxidative stress often occurs as a result of frataxin deficiency [54]. The axonal spheroids arise as a result of various factors including certain cytoskeletal changes, impaired axonal transport, and autophagic flux [54,55,56,57]. Shorter dendrites due to changes in microtubules dynamics are also a result of neurites been affected by oxidative stress damage [56,58,59]. Subsequently, axonal growth can be enhanced by the reduction of oxidative stress in affected neurons. This could be via the direct introduction of antioxidants or via drugs that improve Nrf2 expression [56,60]. These changes negatively impact the cellular viability of neurons and glia, thus precipitating cell death via apoptosis or autophagy [55,61,62,63,64]. Iron plays a major role in inducing cell death. Iron-dependent induction of cell death is a process known as ferroptosis and is associated with an increase in ROS and lipid peroxidation alongside a reduction in GSH [65]. 

FRDA is a disorder involving several systems affecting both the central and peripheral nervous systems, the musculoskeletal system, the myocardium, and the endocrine pancreas. Even though the classical FRDA phenotype exhibits much variation, gait and limb ataxia, dysarthria, and loss of lower limb reflexes with deep sensory loss are always present. Symptoms tend to appear between the ages of 10 and 16, and the mixed ataxia associated with the disease results from peripheral sensory neuropathy, spinocerebellar tract degeneration, and cerebellar pathology. Dysarthria progresses from slow, slurred speech at the onset of disease to unintelligibility in the advanced stages. Pyramidal weakness, particularly of the lower limbs, and distal wasting additionally exacerbate disability occurring in later stages. Dysphagia is common and worsens with disease progression. Sensorineural deafness has been known to develop in certain cases [53]. Frequent oculomotor abnormalities in FRDA include fixation instability with frequent square-wave jerks, while gaze-evoked nystagmus occurs less frequently. Approximately two-thirds of patients display clinical or subclinical optic neuropathy [66,67]. Scoliosis, pes cavus, and talipes equinovarus are some of the more common musculoskeletal abnormalities [53]. Subtle cognitive deficits involving various domains, including executive function, speed and attention, working memory, and visuospatial reasoning, have been noted [68]. In 2017, Nieto et al. demonstrated that both somatic–motivational and cognitive–affective symptoms of depression commonly occur in individuals with FRDA. Additionally, symptoms of depression may exacerbate cognitive and affective symptoms [69]. FRDA is strongly linked to cardiomyopathy, and it is thought that cardiac wall abnormalities are present in most patients, albeit most often, these are asymptomatic. A younger age at disease onset and a longer disease duration increase the risk of DM, which can present acutely with ketoacidosis. Evidence indicates that both insulin deficiency resulting from β-cell apoptosis and insulin resistance lead to glucose intolerance and eventually DM [53]. The diagnosis of FA is mostly reliant on history and physical examination. If a suspicion of FA arises, subsequent tests should be performed: genetic testing (a trinucleotide repeat expansion assay is available, FA is the only disease with pathological GAA) is advised, and MRI is the preferred test for assessment of the extent of atrophic changes. Patients exhibiting the likelihood of having FA should have an MRI of the brain and spinal cord, the findings of which will show atrophy of the cervical/thoracic spinal cord and cerebellum. Examination of common symptoms consists of the following tests: an electrocardiogram, echocardiogram, auditory testing, and vision testing [70]. Although the disease was identified over 150 years ago, a cure for FA has yet to be found. At present, treatment is based on the management of symptoms and complications such as diabetes mellitus and heart failure [53]. Nonspecific Fe chelators (e.g., desferrioxamine) for the specific reduction of mitochondrial Fe overload are most likely ineffective; a clinical trial was halted due to lack of efficacy [71]. An ideal treatment option for FRDA could be based on the provision of adequate amounts of frataxin to patients, thus leading to improved protein function. This supplementation can be carried out through gene therapy, transplantation of hematopoietic stem and progenitor cells, or enhancement of FXN transcription [72,73,74,75]. DFP (1, 2 dimethyl-3-hydroxy-pyrid-4-one), a lipid soluble iron chelator that can be orally administered is regarded to be the prototypic compound for iron redistribution [76]. Studies carried out over recent decades have researched the potential for the treatment of neurodegenerative diseases associated with localised elevation of iron levels via the use of certain molecules that present pharmacophore characteristics for iron chelation [77]. These molecules are derivatives of 8-hydroxyquinoline, hydroxypyridone, and arylhydrazones. Some of these compounds display the ability to selectively reduce excess iron levels in mitochondria [78]. A new compound named CT51 (N-(1,3-dihydroxy-2-(hydroxymethyl)propan-2-yl)-2-(7-hydroxy-2-oxo-2H-chromen-4yl acetamide) has recently been synthesised [79]. CT51 is an extremely selective iron chelator and exhibits a considerable capability for eliminating free radicals and as such reducing oxidative damage to cells. These drugs might enhance the effects of current pharmacological treatments and increase the quality of life while slowing disease progression. However, they are not a curative strategy, as they do not eliminate frataxin deficiency [65]. There is no evidence for treatments to slow or reverse the neurodegeneration associated with FRDA. More treatments being tested are rationally designed and are aimed specifically at pathways based on the understanding of how *FXN* mutations lead to disease [80].

## 4. Manganese Homeostasis and Neurotoxicity

Under homeostatic conditions, Mn is dispersed through portal circulation in either a passive manner involving diffusion or is actively transported thanks to transmembrane transporters. A number of transporter proteins enable Mn penetration of the BBB. Mn transport into the brain occurs with the help of a few carrier proteins, such as DMT1, zinc-collaborating protein 8 (ZIP8, encoded by SLC39A8) and ZIP14 (SLC39A14), transferrin and TR, citrate carrier, and calcium channels, in the form of Mn^2+^, Mn-citrate, or Mn^3+^-transferrin. All the vehicle components known to date have an influence in conveying Mn to the cerebrum, and it is improbable that the flow of Mn across the BBB can be ascribed to a solitary transporter [81].

Mn neurotoxicity is related to high concentrations of Mn in different areas of the brain, including the basal ganglia, frontal cortex, and cerebellum [81,82].

Mn stimulates oxidative stress through the excessive production of ROS and neuroinflammation via the elevation of proinflammatory cytokines, thus showing a critical connection between oxidative stress, inflammation, and Mn-induced neurotoxicity [81,83,84,85,86]. Glial cell activation plays a significant role in inducing Mn neurotoxicity via stimulation of the release of non-neuronally derived ROS and inflammatory mediators such as proinflammatory cytokines. The stimulation of microglia plays a significant role in the response to environmental stress and immunological challenges by scavenging neurotoxins, thereby eliminating dying cells and cellular waste and releasing proinflammatory cytokines [87]. Along with the information that excessive concentrations of Mn induce oxidative stress, its role in maintaining superoxide concentration shows Mn to be a critical component of the redox interface between an organism and its environment [88].

Additionally, Mn-induced neurodegeneration is regulated by the initiation of several transcription factors, including the expression of human nuclear factor erythroid-derived 2-like 2 (NRF2), which is involved in mitochondrial biogenesis and antioxidant reactions [8,89,90]. Mn’s capacity to impede various neurotransmitter systems has been extensively reported. Mn influences different components associated with the regulation of neurotransmitters such as GABA (*gamma*-aminobutyric acid), glutamate, dopamine, norepinephrine, serotonin, and Ach [91]. This shows that Mn toxicity can influence multiple brain functions connected to neurotransmitters, resulting in issues such as motor, learning, memory, and cognitive deficits.

While an immediate link between Mn toxicity and Parkinson’s disease-related Lewy bodies has yet to be established, research has shown that Mn disrupts the function of α-synuclein (αSyn), which is a protein normally present in Lewy bodies in the brains of Parkinson’s disease patients. More research also indicates that Mn is related to Alzheimer’s disease through the excessive production and accumulation of amyloid-β (Aβ) [81].

### 4.1. Neuropsychiatric Diseases Associated with Manganese Toxicosis

#### 4.1.1. Manganism

Manganism is distinguished by neuronal loss and reactive gliosis in the globus pallidus and substantia nigra pars reticulata (SNpr) in the absence of Lewy bodies (the protein aggregates typical of Parkinson disease. Symptoms of Mn neurotoxicity may be delayed up to 1–2 years after exposure and thereafter progress slowly [4,92,93,94].

Manganism was first described by Couper 1837 (Couper J. 1837). Patients with manganism exhibit a number of symptoms. These include hypophonia, inordinate salivation, mental, cognitive, and behavioural impediments, and motor disorders (muscle weakness, limb tremor, and a bent posture while walking). The motor and behavioural effects of Mn toxicity are considered permanent, and research shows that further progression is possible even after cessation of chronic exposure. Various compounds exhibiting neuroprotective characteristics and therapeutic means have been researched to assess their effectiveness in protecting against Mn neurotoxicity while taking into consideration the pharmacokinetics and Mn-related toxicity mechanisms [8].

The phenotype of manganism can be characterised based on the prevalent symptoms: (1) behaviour changes; (2) parkinsonian features; and (3) dystonia with severe gait disturbances, i.e., the “cock walk”, in which patients walk on their toes, with elbows bent and the spine straight. The early phase of psychiatric symptoms is also described as “manganese madness” or “*locura manganica*”. It is distinguished by emotional instability, irritability, mood swings, attention disturbances or delayed response times; mania, impulsive or violent behavior, hallucinations, sleep and eating (anorexia) disorders, as well as sexual disturbances while exhibiting few or subtle motor effects. A later stage (‘established’ stage) is characterised by motor symptoms such as bradykinesia and rigidity with slight resting tremor, speech disturbances, masked face, and postural unsteadiness. Walking disturbances can be followed by dysarthria and deteriorating handwriting, as well as axial and extremity dystonia [4,91]. Other manifestations include hypomimia, rigidity, disturbed speech, bradykinesia, and walking difficulty, which are consistent with symptoms of idiopathic Parkinson’s disease [91,95]. In 2019, Shweta Prasad et al. reported a unilateral tremor without obvious parkinsonism in patients with Mn toxicity. As such, manganism should be evaluated and excluded in those who are at risk of Mn exposure [96].

The dissimilarities between Mn-induced parkinsonism and Parkinson’s disease have been emphasised in many review works. In contrast to typical PD, Mn-induced parkinsonism does not respond to levodopa treatment and clinically manifests without resting tremor and more frequently early gait disorders with dystonia [91,97,98].

The diagnosis of Mn poisoning depends strongly on the neurological manifestations, occupational exposure history, MRI brain appearances, and presence of Mn in biological samples. At present, there are no established biomarkers of cumulative Mn exposure, and there are no prognostic biomarkers of its neurotoxic effects. Several potential biomarkers are used in the evaluation of Mn exposure in children and are found in maternal/cord blood, blood, serum, plasma, urine, nails, saliva, and hair [99]. Blood and urinary Mn levels indicate exposure over a brief and recent amount of time (over a period of hours to days), whereas nails and hair indicate extended periods of exposure up to several months [99,100,101]. Nails, particularly toenails, show higher levels related to longer-term exposure [102]. Hair Mn is considered the most reliable and valid biomarker for assessing exposure in children and has been found to be linked with a decrease in the intelligence quotient (IQ) in most studies [103]. Hair grows at an average rate of 1 cm per month and can provide information regarding exposure for a period of 1–6 months, taking into consideration variability as a result of differences in hair pigmentation and potential external contamination [101,104].

A recently discovered biomarker, Mn in primary teeth, can assess exposure during prenatal development and early childhood. The deposition of metals in teeth has been discovered to be related to exposure in the pre- and postnatal periods by measuring Mn in house dust and in the blood and bone of the mother prenatally as well as cord blood and blood in the postnatal period [105,106]. This biomarker can supply information about the period and intensity of exposure during the foetal period, with particular regard to the second and third trimesters, and during early childhood and cumulative early-life exposure [101,107].

Due to its paramagnetic properties, Mn is highly visible in MRI imaging. Mn accumulates preferentially in the globus pallidus of the basal ganglia, where it creates high signals on brain MRI. These hyperintensities are bilateral, symmetrical, and visible in T1-weighted MRI [108]. A pallidal index (PI) can be computed to quantify Mn intensity by dividing the signal observed in the globus pallidus by the signal observed in the white matter in the frontal cortex and multiplying the result by 100. PI has been shown to be a dependable marker for Mn exposure [109,110]. People with over 5 years of work experience with Mn exposure exhibited almost a 100% occurrence of enhanced PI, suggesting that PI is specific for Mn exposure even in the absence of clinical manifestations. A disadvantage of the use of MRI is that its use is limited to the detection of recent exposure. In studies involving smelters or intravenous ephedrone users, the signal in the globus pallidus vanishes almost entirely 5–6 months after exposure cessation [95,110]. Magnetic resonance spectroscopy (MRS) is another useful method of quantifying neurochemical markers associated with Mn exposure. The quantitation of GABA, glutamate, total creatine (tCr), and *N*-acetyl-aspartate (NAA)/tCr values, along with other macromolecules, is now possible thanks to MRS. In the thalamus and basal ganglia of Mn-exposed smelters, levels of GABA concentration were increased twofold, even while the mean airborne Mn level was only 0.18 mg/m^3^, which is a level below standard occupational norms. This could mean that an early metabolic or pathological change occurs even with low-level Mn exposure, and MRS appears able to detect these early biochemical changes before the manifestation of full-blown symptoms [95,109].

Treatment of manganism toxicity comprises the treatment of the immediate dangers of toxicity and the management of symptoms caused by extended exposure. The most readily available form of treatment for manganism is exposure cessation regardless of source, be it occupational, environmental, or iatrogenic [111].

Levodopa is regarded as ineffective in the treatment of manganism. In addition, after the cessation of L-dopa use, patients experienced further progression of disease despite an early start to therapy [111,112].

Chelating therapy with intravenous ethylenediaminetetraacetic acid (EDTA) has been successfully demonstrated to increase the elimination of Mn via urine and decrease Mn concentrations in blood [4,95]. Even so, EDTA molecules are extremely water soluble and penetrate the blood–brain barrier poorly. Due to this low brain bioavailability, the effectiveness of EDTA in the treatment of Mn intoxication is limited [113].

Another chelation molecule, para-aminosalicylic acid (PAS), normally utilised in the treatment of tuberculosis, has demonstrated clinical benefit after use in patients with manganism. PAS and its metabolites accumulate within the choroid plexus, brain parenchyma, and CSF, thus making it an ideal chelator for treatment use [95].

Fe supplementation is an additional form of treatment. Iron is a competitive inhibitor of Mn intestinal absorption [114]. In a case of congenital hypermanganesaemia, a mix of Fe supplementation and chelation therapy proved effective in effectively lowering blood Mn levels and thus reduced the body Mn load, prompting major improvements in neurologic symptoms [4,115].

Newer treatment methods currently being researched include the use of taurine and rasagiline. Taurine use reduces the toxic effects of Mn in vitro, mainly via conservation of mitochondrial functionality in CNS tissues [116,117]. Taurine also seems to improve the learning and memory impairments related to chronic manganism [118]. Rasagiline is a monoamine oxidase inhibitor (MAO-I) used to inhibit dopamine metabolism in patients with Parkinson’s disease. Rasagiline seems to offer benefits due to the protection it offers from reactive oxygen species created as a result of Mn toxicity [111,119].

#### 4.1.2. Attention-Deficit/Hyperactivity Disorder

Attention-deficit/hyperactivity disorder (ADHD) is a psychiatric condition that is known to impede children’s ability to function. ADHD affects approximately 6.8% of children, 2.8% of adolescents, and 2.5% of adults worldwide [120]. Sufferers of this disorder exhibit developmentally inappropriate levels of inattentiveness, hyperactivity, or impulsivity. While in the past there were two different diagnoses of Attention Deficit Disorder vs. Attention Deficit Hyperactivity Disorder, the DSM-IV has now combined the two into one disorder with three subtypes: predominantly inattentive, predominantly hyperactive, or combined type. The symptoms normally begin at a young age and generally include a lack of attention, lack of concentration, disorganisation, difficulty with task completion, forgetfulness, and loss of personal items. These symptoms need to have been present before the age of 12, have lasted six months, and interfere with everyday activities in order to be labelled as “ADHD”. These symptoms must be evident in more than one setting (i.e., at home and at school or at school and at afterschool activities). The disorder can have significant and far-reaching effects, including and not limited to poor social interactions, increased risky behaviours, job loss, and difficulty with schoolwork.

ADHD must be assessed within the context of what is developmentally and culturally appropriate for a person. It is seen as a dysfunction of executive functioning, which is mainly driven by frontal lobe activity. As such, patients with ADHD display impairment regarding not only attention and focus but also decision making and emotional regulation. Children with ADHD may find social interactions difficult, may be easily frustrated, and could be impulsive. They are often seen to be troublesome [121]. Regrettably, the objective assessments available for ADHD, including neuropsychological tests (which have a low strength of evidence) as well as EEG and neuroimaging (for which the evidence remains insufficient), are of little use in shedding light on the diagnosis [122].

While the precise pathogenesis of ADHD is still unknown, research shows that individuals with ADHD have smaller brain volumes in regard to particular regions such as the prefrontal cortex and basal ganglia or have experienced greater exposure to environmental pollution, for example, in the form of lead or polybrominated diphenyl ethers [123]. One of the most well-known theories of the pathogenesis of ADHD is the “dopamine hypothesis” [124], which attributes ADHD to dysregulation of the dopaminergic system. Mn’s capacity to impede various neurotransmitter systems has been extensively reported. Mn influences different components associated with the regulation of neurotransmitters such as GABA, glutamate, dopamine, norepinephrine, serotonin, and ACh [91]. 

Mn impedes neurotransmitter regulation by hindering the enzyme activity that controls ideal neurotransmitter levels. Significant concentrations of glutamate and/or acetylcholine in the synaptic cleft cause excess stimulation of NMDA receptors, thus prompting excitotoxic neuronal death. Mn can be transferred into dopaminergic neurons via DAT. Surplus cellular Mn^2+^ impedes Ca^2+^ homeostasis in cells, which leads to a reduced production of dopamine and neuronal death. Mn also causes mitochondrial stress, prompting neuronal apoptosis and/or gliosis [87].

A review article demonstrated that ADHD patients have decreased access to dopamine receptor isoforms and increased levels of dopamine transporter (DAT) binding in comparison with controls [125]. The most commonly used medication for ADHD, methylphenidate (and its derivatives), is believed to be effective due to its ability to block DAT and promote dopamine release [124,125]. Other factors referenced in the aetiology and pathology of ADHD include genetic factors [126], interaction of genes and nutrition [127], epigenetic [124,128], and environmental elements, together with stress. Nutrition and diet were also investigated as potential influencing factors, as restriction and elimination diets have been tried in ADHD treatments [129]. As potential causes of ADHD in children, oxidative stress, exposure to metal toxicity, decreased methylation of relevant genes, cerebral hypoperfusion, and mitochondrial dysfunctions have been listed. The gut microbiota has been late connected with dietary patterns and linked to susceptibility to ADHD [130,131,132]. Jörg Schullehner discovered that increasing exposure to higher levels of Mn in drinking water was linked with an increased risk of the ADHD-inattentive subtype but not the ADHD-combined subtype [133].

The primary categories of ADHD treatment include pharmacologic and non-pharmacologic treatments, such as counselling, behavioural, and environmental modification strategies. Each method of treatment has been demonstrated to be effective; however, a combination of various treatment types has been shown to be most effective [134].

Behavioural therapy and parent behavioural training could possibly tackle primary symptoms and functional deficiencies that are prevalent in children with ADHD. Pharmacological treatment can be beneficial in the management of ADHD core symptoms by reducing distractibility, improving sustained attention, reducing impulsive behaviours, and improving activity levels, all of which lead to improved performance across all settings. Pharmacologic agents used to treat ADHD can be divided into two main classes: stimulant and non-stimulant medications [134].

Behavioural interventions focusing on behavioural modification, such as parent behaviour-management training and school behaviour-management programs, have thus far been shown to be highly effective. Other forms of intervention, such diets, herbal and other types of supplements, EEG training, and neuropsychological or cognitive training interventions, are lacking in proven efficacy in comparison to levels achieved by the US Food and Drug Administration-approved ADHD medications and the previously mentioned behavioural interventions.

In the Multimodal Treatment of ADHD (MTA) study, funded by the National Institute of Mental Health, the benefits of both stimulant medication and behavioural interventions in a multisite study over a 14-month period, with 10-year follow-up surveillance, were researched. Even though stimulant pharmacological treatment had the strongest effect on core ADHD symptoms and behavioural trainings were most acceptable to families, the combination of medication and behavioural therapy was most effective, particularly when comorbidity or additional family issues were involved. However, subsequent to the end of the active trial, participant parties no longer received a similar level of care. As such, in light of the fact that neither medication nor behavioural treatment of ADHD is curative and that both work only when being actively administered, it has been challenging to demonstrate their lasting benefits [135].

As previously mentioned, the conventional treatment for ADHD includes behavioural and educational interventions, which are often in addition to psychostimulant medication. The psychostimulants MPH and dextroamphetamine and the non-stimulant prefrontal cortex noradrenaline reuptake inhibitor atomoxetine are regarded as standard treatments for ADHD. MPH is a central nervous system stimulant, thus increasing attentivity while reducing hyperactivity and impulsivity by impeding the reuptake of dopamine in the striatum without setting of its release. Nevertheless, neurotransmitters, while aiding synaptic communication, are also involved in central nervous system (CNS) development, including morphogenesis and the proliferation, migration, and differentiation of neurons. Subsequently, any interference with neurotransmitter systems possibly produces acute and/or long-lasting changes to CNS structure and function. Indeed, neurotoxicants associated with ADHD have been found to impair dopaminergic neurons. It is also thought that dopaminergic and noradrenergic dysfunction trigger ADHD behaviour, as the volume and activity of these brain areas, typically involved in attention, emotion, and behaviour, are decreased in patients.

Additionally, while ADHD medications do reduce symptoms in the majority of patients, most of these patients are likely to experience side effects within the first 6 months of MPH use. A review reports undesirable effects, such as insomnia and decreased appetite, in approximately 25% of patients using MPH. Other side effects range from weight loss, abdominal pain, sleep disturbances, headaches, irritability, and depressed mood and appetite to reports of stimulant-induced psychosis. MPH is prescribed for prolonged usage to a large number of ADHD patients but has been linked to possible publication bias regarding reported efficacy. Furthermore, parents are often unwilling to use MPH due to negative publicity surrounding its use and its frequent side effects, and as a result, non-compliance with therapy is high. Additionally, pharmacotherapy may be incapable of normalising functioning due to the adaptation of the mind and body to the medication, resulting in a situation where a large number of patients no longer experience any difference in symptoms. A possible part of the efficacy of the medication could be a result of the extra attention being given to the child or a placebo response [131].

#### 4.1.3. Ephedrone Encephalopathy

Ephedrone encephalopathy is an uncommon ailment linked to the abuse of methcathinone. Methcathinone, also known as ephedrone, is a psychostimulant that can be manufactured at home using over-the-counter medications containing pseudoephedrine and potassium permanganate. Excessive accumulation of ingested Mn results in damage to the basal ganglia [136]. Abuse of a homemade version of the psychostimulant drug methcathinone (Mcat) can result in prolonged and incapacitating extrapyramidal syndrome [137]. Ephedrone encephalopathy comprises a group of symptoms resulting from Mn deposition within the central nervous system (CNS) due to the abuse of ephedrone (methcathinone), which is obtained via reactions involving excessive amounts of Mn-containing oxidants. The diagnosis is made based on the contrast-enhanced brain MRI results characteristic of this syndrome, clinical manifestation, and history of ephedrone abuse [138].

The first symptoms develop within 3–14 months of regular usage. Patients are most likely to complain of postural instability and falls, psychomotor speed decline, and speech disorders. Then, the face becomes hypomimic—that is to say, it becomes mask-like. Speech disorders are similar to hypokinetic dystonic dysarthria in nature. “Ephedrone speech” is soft and dysphonic, unlike speech as a result of supranuclear palsy or Parkinson’s disease treatment by electrical deep brain stimulation of the subthalamic nucleus (DBS-STN) [136]. Symptoms of dystonia and parkinsonism are the primary clinical features of the disease.

Neuropsychiatric symptoms, known in the past as “manganese madness”, most commonly include apathy, depression, and mild cognitive impairment in the form of attention disorders and verbal and visual working memory process impairment [139,140].

In 30% of cases, the disease progresses further, leading to significant disability despite discontinuation of methcathinone usage. Thus far, all treatments have failed to produce the desired results.

The primary diagnostic test in the diagnosis of ephedrone encephalopathy is magnetic resonance imaging (MRI). A characteristic feature is the amplification of the signal bilaterally in T1-weighted images in the basal ganglia—mainly within the pale inner knob, shell, caudate nucleus, and black substance. The lesions are similar to other damage lesions linked to Mn accumulation [141]. There was no correlation between the severity of changes in MRI and the duration of intoxication or the combined dose ingested. Additionally, the radiological picture does not correspond to the level of clinical severity of the disease. The MRI findings gradually vanish over 9–18 months following the discontinuation of methylcathinone substances. Thus far, no effective therapy has been discovered [136].

#### 4.1.4. HMNDYT1-SLC30A10 Deficiency

In 2012, hypermanganesaemia with dystonia 1 (HMNDYT1) as a result of bi-allelic mutations in SLC30A10 became the first inherited Mn transporter defect to be described [142,143]. Systemic Mn accumulation causes a syndrome comprising several symptoms and ailments, such as hypermanganesaemia, polycythaemia, dystonia, chronic liver disease (ranging from asymptomatic steatosis to cirrhosis with liver insufficiency), and depletion of Fe stores. Blood Mn levels are substantially elevated, often as high as ten times the normal level. In brain MRI, the accumulation of Mn is apparent in the basal ganglia, especially the globus pallidus and striatum, with evident hyperintensity on T1-weighted imaging and corresponding hypointensity on T2-weighted imaging [144,145,146,147,148]. There is also involvement of the white matter of the cerebrum and cerebellum, midbrain, dorsal pons, and medulla with a characteristic exclusion of the ventral pons. Clinically, most patients develop dystonia during early childhood. Lower limb dystonia causes a pathognomonic high-stepping gait, which is also known as the “cock walk gait”. White matter involvement can lead to the development of spasticity and pyramidal tract signs. A late-onset form that appeared as adult parkinsonism unresponsive to L-DOPA treatment was also reported in one family. The accumulation of Mn in the liver is hepatotoxic and ultimately causes liver disease. However, at the time of disease presentation, the liver is often clinically uninvolved. Liver disease ranges from mild forms such as steatosis to severe forms such as cirrhosis. Polycythaemia has been reported in all patients and can be present before the onset of clinical symptoms [114,142]. Chelation with EDTA-CaNa_2_ has been successfully used to lessen Mn accumulation, treat neurological symptoms, and halt liver disease progression. In the majority of cases, Mn chelation leads to a reduction in polycythaemia, normalisation of Fe parameters, and stabilisation of blood Mn levels. EDTA-CaNa_2_ was given intravenously over 5 to 8 days every 4 weeks. Careful monitoring of calcium and other trace metal levels, such as Zn, Cu, and selenium (Se), is essential to avoid unwanted side effects [149]. Some studies suggest that 2,3-dimercaptosuccinic acid and d-penicillamine are also effective oral alternatives [145,150]. Iron supplementation has additionally been proven to reduce clinical symptoms to a certain degree and lower Mn levels [147].

#### 4.1.5. HMNDYT2-SLC39A14 Deficiency

In 2016, bi-allelic mutations in SLC39A14 were detected in individuals presenting with characteristic features of Mn neurotoxicity, such as rapidly progressive dystonia with varying signs of parkinsonism and T1 hyperintensity of the globus pallidus in brain MRI. While hypermanganesaemia was found in individuals with HMNDYT2, they did not show systemic features of Mn overload, such as liver disease or polycythaemia. Blood levels of Fe, Zn, and cadmium (Cd), which are divalent metals able to be transported by SLC39A14 in in vitro assays, were normal. Liver MRI was also normal, thus indicating the absence of hepatic Mn accumulation. In general, the onset of neurological symptoms seems to occur earlier than that observed in individuals with HMNDYT1, with some people being severely affected by hypotonia and dystonia within the first year of life [143]. Then, axial hypotonia is succeeded by dystonia, spasticity, dysarthria, bulbar dysfunction, and other features of parkinsonism [148]. MRI brain appearances are similar to those seen in HMNDYT1. Anti-spasticity medications (baclofen and botulinum toxin) and L-dopa have had partial success. Although chelation therapy with intravenous administration of disodium calcium edetate early in the disease course shows certain promise, further studies are required. In addition to Mn chelation, dietary restriction of Mn could be beneficial [148].

#### 4.1.6. CDG2N-SLC39A8 Deficiency

The first inherited disorder related to Mn deficiency and caused by bi-allelic mutations in SLC39A8, a Mn uptake transporter, was identified in 2015 [151,152]. Systemic Mn deficiency results in a large number of varied symptoms, such as developmental delay, intellectual disability, failure to thrive, short stature or dwarfism, hypotonia, dystonia, strabismus, seizures, cranial asymmetry, and deafness. Typically, blood Mn levels are low. Patients have been known to additionally present with Leigh-like mitochondrial disease characterised by elevated levels of CSF lactate and abnormal respiratory chain enzymology associated with hyperintensity of the basal ganglia on T2-weighted MR imaging. MRI brain imaging is not characteristic; most patients demonstrate cerebellar and/or cerebral atrophy [151,152,153]. Oral Mn supplementation has been shown to be an effective treatment method, particularly when preceded by galactose supplementation. This results in stabilisation of the glycosylation pattern [148,151].

#### 4.1.7. Hepatic Encephalopathy (HE)

Hepatic encephalopathy is a complex of potentially reversible neurological and psychiatric disturbances resulting from hepatic failure and/or portal hypertension, the clinical spectrum of which ranges from poor attentional concentration to coma.

The pathogenesis of HE is still not fully understood. The most common pathological mechanism taken into account is impaired neurotransmission due to metabolic changes caused by liver failure, inflammatory responses, and changes in brain energy metabolism or in the blood–brain barrier. In the case of a high concentration of ammonia in the blood, its penetration into the CNS tissue is so great that the defense mechanisms fail, and this compound has a toxic effect on the brain. There are other factors that cause hepatic encephalopathy, including short-chain fatty acids, methionine, and its derivatives, mercaptans. It is often emphasised, as mentioned before, that the impaired ability to remove ammonia and other products of metabolism of nitrogen compounds from the blood and swelling of astrocytes as consequence of hyperammonaemia is a key in the development of HE in patients with cirrhosis [154]. Regarding toxicosis in HE, impaired hepatic removal can cause Mn deposition in the brain, leading to oxidative/nitrosative stress, glutamate receptor-mediated excitotoxicity, and neuroinflammation [155,156,157]. Mn may act synergistically with ammonia in the promotion of Alzheimer type II astrocytosis, increase glutamine accumulation, and inhibit astrocytic glutamate uptake and the production of excess free radicals [158,159]. Mn in the central nervous system can cause astrocyte mitochondrial dysfunction and can affect cortical astrocytes, leading to decreased high affinity transport of glutamate and an increase in extracellular glutamate concentrations, consequently resulting in excitotoxicity/nitrosative stress-mediated neuronal cell damage [156]. Mn concentrations are elevated in the blood and postmortem brain tissue of cirrhotic patients [160,161]. Improvement in liver function is usually connected to regression of HE symptoms, although sometimes, neuropsychiatric changes may become permanent. Clinically, HE is characterised by a number of neurologic and psychiatric symptoms, such as changes in personality, cognitive decline, impaired sleep–wake cycle, and alterations in motor functions. In the beginning, difficult to notice symptoms are a change in handwriting or problems with performing mathematical operations (grade 1). At grades 2 and 3, intellectual functions may be lost, and there may be marked changes in personality. Grades 2 and 3 are easier to diagnose, as severe neurological disorders are present in addition to the above-mentioned symptoms. In very advanced stages, complete loss of consciousness may occur accompanied by cerebral stiffness and unreactive pupils, which is referred to as hepatic coma. However, the most common cause of this disease is hyperammonaemia, the diagnosis of which is the basis for the diagnosis and assessment of the severity of hepatic encephalopathy. High blood urea concentration is associated with the risk of HE [154]. Apart from the possibility of determining the concentration of ammonia in arterial blood, MRI spectroscopic examination of the brain (evaluation of nerve cell metabolism) may be helpful in the diagnosis. Some studies suggest that brain magnetic resonance spectroscopy can help detect minimal hepatic encephalopathy [162]. T1-weighted MRI scans in patients with chronic HE showed bilateral signal hyperintensities in the globus pallidus, which has been linked with Mn deposition [162,163]. The hyperintensity of T1 in HE patients was correlated with a high incidence of extrapyramidal dysfunction, such as tremor, rigidity, and akinesia [164]. For example, the diagnostics also use computed tomography or magnetic resonance imaging (helpful in identifying cerebral oedema or other pathological conditions). EEG can show a decline in basal function in the early stages of HE, focal or generalised slow waves, three-phase waves, and paroxysms of spikes or slow waves. In advanced stages of HE, a reduced alpha rhythm may appear with an increase in delta activity.

The goal of HE treatment is to reduce ammonia absorption from the intestinal lumen with the use of lactulose 25 mL twice a day or lactitol. Protein restriction is advised. Intravenous L-ornithine–L-aspartate also lowers ammonia levels and can be used as an alternative in patients who do not respond to lactulose [165]. In patients with recurrent HE with cirrhosis, rifaximin (550 mg twice a day) can be added to lactulose to alter gut microbiota. No nutrition recommendations are made concerning Mn reduction [166].

## 5. Neuropsychiatric Diseases Associated with Other Metal Toxicosis

### 5.1. Prion Disease and “Prion-Like Disease”—Metal Overload

#### 5.1.1. Transmissible Spongiform Encephalopathies—Prion Disease

Human prion diseases are mostly classified into inherited, sporadic, and acquired types. Accumulating evidence suggests that exposure to environmental metals is a risk factor for the development of prions and that metal ions can directly bind to prion and prion-like proteins, which affects the amount of amyloid aggregates [167,168,169,170]. Prion [167,168] protein is associated with transmissible spongiform encephalopathies (TSEs). Transmissible spongiform encephalopathies are a kind of neurodegenerative disease caused by the accumulation of a harmful isoform of the prion protein known as scrapie prion protein PrPSc [167]. PrPSc has the ability to precipitate in insoluble and protease-resistant amyloid aggregates and can cause neuronal cell death. Cellular prion protein (PrP^C^) may have the affinity to bind metals [171]. There is growing evidence that excess Mn has been found in the blood and brains of humans with Creutzfeldt–Jakob disease (CJD), bovines infected with bovine spongiform encephalopathy (BSE), and scrapie-infected mice [38,42]. There is also some evidence that Cu may have a protective role in the regulation of PrP–Cu interactions and can modulate protection against Cu^2+^-mediated oxidative stress, Cu transport, and Cu-dependent cellular signaling [172]. Available data on the role of Cu^2+^ in prion disease are still conflicting. Recently, a Cu^2+^-dependent neuroprotective role of PrP^C^ mediated by N-methyl-d-aspartate (NMDA) receptor nitrosylation [173] was demonstrated. In vivo and in vivo experiments showed that the addition of Cu^2+^ induces the conversion of PrP^C^ into PrP^Sc^, increases the infectivity and protease resistance of the protein, and can accelerate prion disease [174]. Cu^2+^ chelation delays the beginning of the disease [170]. Other studies indicate that the presence of Cu^2+^ inhibits the conversion of PrP^C^ into PrP^Sc^ and its accumulation, delaying the onset of the disease in infected cells [169,175]. Moreover, it was demonstrated that exposure to a source of reduced Fe, such as inorganic ferrous chloride (FeCl_2_), induced the conversion of PrP^C^ to PrP^Sc^, implicating a role for this metal in the generation and propagation of PrP^Sc^ [176]. The clinical manifestation is connected with rapid dementia and neurological signs such as cerebellar symptoms (characteristic gait and speech), behavioural disturbances, tremors, myoclonia, and visual disturbances. In vCJD, cerebellar disorders occur in practically all patients, accompanied by symptoms of progressive dementia, psychiatric symptoms (depressed mood, withdrawal, irritability, sleep disturbances), sensory disturbances, tremors, convulsions, swallowing disorders, and urinary and faecal incontinence. Gerstmann–Straussler–Scheinker disease occurs in families with a gene mutation in the gene encoding the cellular prion protein. Initial symptoms include progressive dementia and movement disorders accompanied by seizures, nystagmus, and disturbed vision and hearing. The methods used for antemortem diagnosis are cerebrospinal fluid protein biomarker examination (14-3-3 protein CSF biomarker testing is restricted to patients with clinical symptoms of CJD), electroencephalography and MRI, and real-time quaking-induced conversion (RT-QuIC). In MRI in patients with variant CJD characteristic findings in the posterior thalamic region, the “pulvinar sign” is seen. In sporadic (sCJD) T2-weighted and diffusion-weighted imaging sequences, abnormalities in the cortical grey matter (with cortical ribboning) and deep nuclei in sCJD are detected [177]. Recently, higher levels of neurogranin were found in CJD, and the authors believe that it can play a promising role in CJD prognosis as a marker of neuronal damage [178,179]. The definitive method for prion disease diagnosis is biopsy. No treatment is available.

Many neurodegenerative diseases, such as Alzheimer’s disease, HD, synucleinopathies (with Parkinson’s disease), amyotrophic Lateral Sclerosis (ALS), and frontotemporal dementia, may be included under the definition of “prion-like disease”, as they share some neuropathological features with prion diseases and have metal dyshomeostasis. In the following sections, we will discuss less frequent diseases, such as HD, FRDA, and ALS.

#### 5.1.2. Amyotrophic Lateral Sclerosis

Oxidative stress is recognised as a common element in the pathogenesis of many neurodegenerative disorders, including ALS. Copper homeostasis is disturbed in ALS and may be important for its pathogenesis. In spinal cord tissue taken from patients with sporadic ALS, a significant increase in the amount of Cu was found. Ref. [180] ALS is a motor neuron disease with serious outcomes and an average survival time of 2–5 years after diagnosis. The neurodegenerative process in ALS affects both upper and lower motor neurons. It is a rare disorder and commonly presents in late adulthood. The characteristic symptoms are muscle weakness in the limbs; in some patients, ALS begins in the bulbar muscles, with difficulties in speech and swallowing. ALS shows a number of neuropathological similarities with frontotemporal dementia (FTD), which is why we chose to discuss it in this study. Cognitive impairment is present in 30–50% of patients with ALS and has a negative influence on survival and quality of life in this disease [181]. In 2017, the revised consensus criteria for the diagnosis of frontotemporal dysfunction in ALS were presented [182]. Executive dysfunction with deficits on letter fluency tasks occurs early in the course of the disease. Social cognition deficits are integral to the cognitive profile in ALS, and there are also memory and language deficits and behaviour changes (mostly apathy). Additionally, disinhibition, loss of sympathy and empathy, perseverative, stereotyped, or compulsive behaviour, hyperorality/dietary change, loss of insight (see above), and psychotic symptoms can be seen. The clinical spectra of ALS and FTD are similar, and only the presentation is the opposite [183]. Familial ALS is quite rare (5–10%), and the most intensely studied among several genes responsible for familial ALS (fALS) mutations is the gene encoding Cu,Zn-superoxide dismutase (SOD1) [184]. SOD1 is one of the proteins with the highest affinity for Cu ions in cells, and after several mutations, SOD1 proteins may exhibit distinct affinity for metal binding in vitro, which can be correlated with disease onset or duration in vivo [185]. SOD1 overexpression leads to the abnormal accumulation of Cu ions in the spinal cords of ALS model mice [186]. The level of Cu ions in the mouse spinal cord was elevated in the region most affected by ALS, expressing SOD1 [187]. It was also found that in the spinal cord, the elevation of non-SOD1 Cu levels was highly correlated with disease progression in mice expressing human SOD1 with the G93A mutation (hSOD1G93A) and was not observed in regions less affected than the brain in ALS [187]. This fact can indicate the pathological role of abnormal Cu accumulation in SOD1-ALS cases. Unfortunately, in humans, there is currently no consensus regarding the level of Cu in cases of SOD1-ALS and changes in the proteins maintaining homeostasis of intracellular Cu in cases of ALS. Copper chelators such as trientine, d-penicillamine, and tetrathiomolybdate were used in mouse ALS models to normalise the intracellular Cu levels [187,188,189,190]. Tetrathiomolybdate, a recently developed Cu^II^(atsm) complex, had the highest benefits for transgenic mice expressing mutant SOD1 proteins [187,191]. Modulation of the Cu-binding status of SOD1 in an animal model of ALS can also be used as a therapeutic target [187]. It was also recently shown that in the presence of SOD1–ALS mutations that are able to preserve the ability of the enzyme to bind zinc or Cu, the administration of Cu(II)-diacetyl-bis(N4-methyl-thiosemicarbazone (CuATSM) can be connected with delivery and binding of Cu to SOD1, which can cause proper SOD1 maturation, increased SOD1 activity, and reduced SOD1 aggregation, as well as increased cell viability [192]. The CuATSM complex is a marker of oxidative stress for tissue imaging, and it can be orally bioavailable and blood–brain barrier permeant. Phase I testing of Cu^II^(atsm) in ALS patients was successfully completed [193], and Phase II/III testing is currently underway to establish this compound as a potential therapy for ALS [194].

#### 5.1.3. Huntington’s Disease

HD is a fully penetrant neurodegenerative disease caused by a dominantly inherited CAG trinucleotide repeat expansion in the huntingtin gene on chromosome 4 [195]. The number of CAG repeats increases from the normal range of 16–20 repeats to >35 repeats in patients [196]. Stefania Squadrone et al. found higher levels of the essential elements Fe, chromium (Cr), Se, Zn, and of the nonessential element arsenic in the blood of HD patients. They also discovered that the altered homeostasis of Fe was also correlated by blood analysis, which showed higher values of Fe in HD patients than in controls. Additionally, elevated values of three other essential elements, Cr, Se, and Zn, were noted in patients [197].

Symptoms of this neurodegenerative disease result from progressive motor, psychiatric, and cognitive impairment and include loss of self-awareness and spatial awareness, depression, dementia, and weight loss [198]. Clinically, HD is divided into two forms: juvenile HD (onset of symptoms before 21 years of age and very pronounced clinical symptoms) and late-onset HD (occurring after the age of 60 years). Motor abnormalities such as bradykinesia, chorea, dystonia, and oculomotor symptoms are progressive in nature in HD patients [199]. HD patients also suffer from a certain motor problem characterised by failure to maintain a voluntary muscle contraction at a consistent level. Due to this inability, patients are unable to exert constant pressure during a handshake. This symptom is seen as a characteristic of HD and is called milkmaid’s grip [200]. With the progression of the disease, fewer hyperkinetic movements occur, while bradykinesia and rigidity become more common [201]. Progressive cognitive decline, ranging from early changes in regard to speed of information processing, cognitive inflexibility, and memory retrieval to more severe and widespread symptoms later in the disease course, is typical of the HD phenotype [202]. Patients also display serious psychiatric abnormalities, such as mood swings, depression, and personality changes. Anxiety, obsessive-compulsive disorder, and psychosis are some of the other psychiatric symptoms occurring in the course of the disease [203]. The diagnosis of HD is established on the basis of a confirmed family history or positive genetic test as well as the onset of motor disturbance as defined by the Unified HD Rating Scale (UHDRS) total motor score (TMS) diagnostic confidence score [195]. In 2008, the FDA approved tetrabenazine as the first medication for the treatment of HD-associated chorea. This was followed by the approval of deutetrabenazine as the second medication of chorea linked to HD in April 2017. At present, deutetrabenazine is regarded as an effective therapeutic treatment option for chorea in HD and might present a more favourable adverse effect profile than tetrabenazine. Nonetheless, more studies are required, especially in the form of head-to-head studies comparing deutetrabenazine with other treatment options as well as longer-term clinical experience with deutetrabenazine [204].

#### 5.1.4. Depression

Depression is a heterogeneous disorder that presents in several ways with similar phenotypes but differing aetiologies. Despite the vast number of varying studies focused on the aetiology of depression, no consistent classificatory system has surfaced linking either to the underlying aetiology or indicative of the most likely response to treatment. Several classification methods/subgroups have been used. These include reactive and endogenous depression, melancholia, depression with a seasonal pattern/seasonal affective disorder, atypical depression, and dysthymia. Over time, practical definitions have emerged and have been established in the current two major classification systems, DSM–IV and ICD–10. Practical descriptions of depression have been further divided based on the intensity of symptoms (for example, mild, moderate or severe as found in DSM–IV with regard to major depressive disorder), the longitudinal cause, and symptom clusters of the disorder. Other features of depression, such as reaction to treatment and aetiology, are not included in the classifications and do not have widely acknowledged definitions, even though they are used in clinical practice. In both ICD–10 and DSM–IV, the symptoms must affect functioning to varying degrees that correspond with the episode severity [205]. Treatment of depression includes both pharmacotherapy and psychotherapy.

The current neurobiological theories with the most compelling empirical basis and the highest clinical relevance are evaluated in regard to their strengths and weaknesses. These particular theories are based on studies examining psychosocial stress; stress hormones and neurotransmitters such as serotonin, norepinephrine, dopamine, glutamate and gamma-aminobutyric acid (GABA); neurocircuitry; neurotrophic factors; and circadian rhythms. Since current theories of depression apply only to a certain percentage of patients suffering from depression and not to all, and because pathophysiology may differ with the progression of illness, current knowledge does not favor the concept of a unified hypothesis of depression.

Depressive disorders could be provoked by neurochemical changes occurring in response to stressful events. Changes involving neuronal plasticity and neuronal survival may also play a role in depression alongside neuroendocrine substrates (e.g., corticotropin-releasing hormone) and neurotransmitters (serotonin and GABA) [206]. Different minerals are thought to be linked to different mechanisms of disease pathophysiology with regard to neurotransmitters. Fe, Cu, and Mn take part in the glutamatergic system, while Cu is also involved in the monoaminergic system. Additionally, Cu and Mn are involved in the GABAergic (GABA: gamma-aminobutyric acid) system [207,208,209].

A decrease in 5-HT levels was reported in rats exposed to a high-Mn diet, thus giving rise to suspicion that 5-HT could be a target of Mn. Lower concentrations of 5-HT and its metabolite in the cortex, hippocampus, and striatum of rats exposed to intranasal administrations of Mn were also noted. Additionally, a decrease in 5-HT in the frontal cortex of rats after intraperitoneal exposure to Mn was reported [210,211]. Modulation of serotonergic neurons leads to a lack of emotional stability and subsequently to anger, depression, sleeplessness, and loss of memory. These symptoms are considered to be early signs of manganism [91,211].

Conversely, two recent cross-sectional studies indicated an inverse correlation between dietary Mn intake and the incidence of depressive symptoms. One study showed that the mean intake level of Mn was lower in school-aged Spanish children with depressive symptoms than in those who did not exhibit such symptoms [212]. Another study carried out among Japanese women indicated that increased Mn intake was associated with a reduced occurrence of depressive symptoms during pregnancy [213].

Oxidative stress pathways may additionally act as triggers in the pathophysiology of depression [214,215]. SOD, an enzymatic antioxidant, is widely distributed in the mitochondrion, nucleus, cytoplasm, and extracellular spaces and plays an important role in human health. SODs are a group of metal-containing enzymes (Cu, Zn-SOD, and Mn-SOD) that speed up the conversion of superoxide anions to hydrogen peroxide. Oxidative stress ensues as a consequence of excessive production of reactive oxygen products or a faulty antioxidant defence system [216]. Previous studies indicated a lower total antioxidant capacity in depressed patients than in controls. Furthermore, lower levels of enzymatic and non-enzymatic antioxidants, including the levels of serum paraoxonase and superoxide dismutase (SOD), were reported in patients suffering from depression than in controls [214,215].

Mlyniec stated that an inverse correlation between Fe and depression is likely; Cu might be positively associated with depression, while Mn could either be negatively or positively connected by means of the neurotransmitter systems involved in the pathophysiology of depression [207,208]. More observational studies and randomised clinical trials are required to further explore the connection between Mn deficiency/overexposure and depressive disorders [209].

## 6. Conclusions

Fe and Mn are required for proper physiological functions in the brain, but they also play important roles in in the pathology of the psychoneurological disorders mentioned above. There is a limited number of studies investigating the amount of Fe, Cu, and Mn in the brain of patients afflicted by the psychoneurological disorders mentioned above. Understanding the impact of Fe/Cu/Mn on these diseases is the only first step in their diagnosis. Most of disorders with metals accumulation have symptoms of damage to the extrapyramidal system or cerebellum such as dystonia, ataxia, and tremor. They are often accompanied by psychiatric symptoms, but these are non-specific, and they may also precede the neurological symptoms. 

Next, it will be important to investigate whether these patients have elevated metals in their brains by radiological methods such as MRI. However, changes cannot always be found on baseline neuroimaging. In this paper, we present disease-specific MRI images to guide the reader in making an appropriate diagnosis. It is also very important to find novel pharmacological modalities to contribute to these psychoneurological disorders. There is still a debate among researchers as to whether chelator therapy is beneficial or harmful to those patients. Chelators also remove essential trace metals during treatment.
